# Influence of alcohol and other substances of abuse at the time of injury among patients in a Norwegian emergency department

**DOI:** 10.1186/s12873-016-0085-2

**Published:** 2016-06-08

**Authors:** Eirin Bakke, Stig Tore Bogstrand, Per Trygve Normann, Øivind Ekeberg, Liliana Bachs

**Affiliations:** Division of Forensic Sciences, Norwegian Institute of Public Health, PO Box 4404, Nydalen, 0403 Oslo Norway; Department of Acute Medicine, Oslo University Hospital, 0407 Oslo, Norway

**Keywords:** Substance influence, Alcohol impairment, Injury, Emergency department

## Abstract

**Background:**

The presence of alcohol or other substances of abuse in blood or urine from injured patients is often used as a proxy for substance influence at the time of injury. The aim of this study was to obtain an estimate of substance influence at the time of injury based on blood concentrations of alcohol and other substances of abuse, and to explore the relationship between the substance prevalence at the time of admittance to the hospital and the actual influence at the time of the injury.

**Methods:**

The study included all adult patients admitted to the emergency department of a university hospital during 1 year (*n* = 996). Quantification in blood was done by an enzymatic method for alcohol, and by liquid chromatography-mass spectrometry or gas chromatography-mass spectrometry for 28 other substances of abuse. Concentrations of alcohol and other substances in blood at the time of injury were calculated. The degree of influence was assessed on the basis of the calculated blood concentrations, with a threshold of influence set at a blood alcohol concentration (BAC) of 0.05 %, or a substance concentration leading to an influence similar to that of a BAC of 0.05 %.

**Results:**

A total of 324 patients (32.5 %) were determined to be under the influence at the time of injury. In comparison, 394 patients (39.6 %) had one or more substances above the cut-off limit in blood at the time of admittance to the hospital. Alcohol was the most prevalent substance causing influence at 25.9 %. Among patients with violence-related injuries, almost 75 % were under the influence of alcohol and/or substances. Patients under the influence were younger, and men were more often under the influence than women. More patients were under the influence at nighttime and during weekends than at daytime and on weekdays.

**Conclusions:**

About one third of the injured patients were determined to be under the influence at the time of injury, with alcohol being the most prevalent substance causing influence. Approximately 98 % of the patients with alcohol detected in blood at the time of admittance to the hospital were under the influence of alcohol at the time of injury.

## Background

Alcohol use and use of other impairing substances of abuse increase the risk of injury, as the intake of these substances may lead to psychomotor impairment. Numerous studies have found high prevalence rates of alcohol and other substances of abuse among injured patients treated in hospitals and emergency rooms [1–4]. The prevalence of such substances in blood or urine has often been used as a proxy for substance influence at the time of injury. This rationale, however, has some limitations. To be able to establish a connection between substances of abuse and injuries, actual substance influence must be identified, not only the presence of these substances in blood or urine.

If a study has used urine samples to screen for substances of abuse, the intake may be old and not represent influence at the time of injury. This may also be a problem when using blood samples and low analytical cut-off limits. Compared to urine samples, the presence of alcohol and substances of abuse in blood indicates more recent intake [5, 6], and allows back-calculation from the time of blood sampling to the time of an incident. Further, since the concentration of alcohol and substances in blood will reflect the concentration level in the central nervous system, it can be used to assess the degree of influence based on previous experimental work [7–9]. As most studies on prevalence of substances other than alcohol among injured patients have used urine samples for drug screening, the knowledge of influence by these substances is limited.

For alcohol [10], and to some degree for other impairing substances, the back-calculation of concentrations from the time of blood sampling to the time of an incident is a well-established methodology, and is regularly used in the field of forensic toxicology. Most substances are eliminated according to linear or first-order kinetics, which means that the elimination rate depends on the actual concentration of the given drug in blood. Elimination of alcohol follows non-linear or zero-order kinetics (when the blood alcohol concentration is above 0.02 %). Although interindividual variations in elimination rates of substances as well as of alcohol are considerable, the use of median values gives relevant information about the approximate level of the substance in blood at the time of an incident. The degree to which different substances will influence a person’s behaviour will also vary, but for most substances a positive relationship between blood concentration and influence has been found. For several of these substances, impairment has been experimentally tested with alcohol for comparison as a gold standard. For others, extrapolation is feasible via equipotence tables within drugs of the same class. This procedure is similar to the one used to establish per se limits of drugs in the traffic legislations of several European countries [11].

To be able to assess substance influence among injured patients, an interdisciplinary approach, as well as good clinical data, is needed. Our study engage laboratory investigation and pharmacologic interpretation of blood samples from injured patients, combined with extensive data concerning the involved patients and their injuries, to be able to perform this assessment.

The aim of this study was to calculate the concentration of alcohol and different impairing medicinal and illicit substances at the time of injury, and then assess the degree of influence at the time of injury, in patients admitted to a hospital emergency department due to injuries from accidents or assaults. Furthermore, we wanted to explore the relationship between the substance prevalence at the time of admittance to the hospital and the actual influence at the time of injury.

## Methods

### Setting and subjects

This cross-sectional study was conducted among patients admitted over a period of 1 year to the emergency department of Oslo university hospital at Ullevål due to injuries from intentional and unintentional injuries including those caused by assaults. The hospital is a trauma centre delivering health care to a population of approximately 2.5 million people in the southeastern part of Norway. The data collection comprised blood samples and questionnaires to the patients, as well as hospital records. The patients included were all injured patients over 18 years of age, with the ability to give informed consent. If the patient was not able to give informed consent, his or her next of kin was asked if the patient could participate. A detailed description of the inclusion procedure and patients not included has been given elsewhere [4]. Since the aim of this particular study was to assess the influence of alcohol and other substances of abuse at the time of injury, patients who were admitted solely due to acute poisoning were excluded. To minimize the risk of substance or alcohol intake after the time of injury, we only included patients who arrived at the hospital within 6 h of the injury.

### Measures

Blood samples from all patients were analyzed for the presence of alcohol and other substances of abuse on the Norwegian market which previously have been shown to be associated with increased motor vehicle crash risk [12]. In total, 29 substances including alcohol were quantified at the Division of Forensic Sciences of the Norwegian Institute of Public Health. A detailed description of all substances initially looked for, the cut-off values, and the methods used for identifying the different substances has been reported elsewhere [4]. Findings of drugs given to patients after injury were disregarded. Infrequent drug findings were not included in this study; buprenorphine (2 positive samples), carisoprodol (1 positive sample), dextropropoxyphene (1 positive sample), ethylmorphine (1 positive sample), gammahydroxybutyrate (1 positive sample), meprobamat (1 positive sample), oxycodone (1 positive sample) and phenobarbital (2 positive samples). Carbamazepine was not included, despite 10 positive samples, since all the concentrations of this substance were in the therapeutic range, and not considered to contribute to the degree of influence. None of the patients included tested positive for phenazepam or 6-monoacetylmorhine (representing heroin intake). All substances included in this study and the cut-off values are given in Table 1.

**Table 1:** Substances included, cut-off limits and concentration groups used to estimate degree of substance influence

Drugs	Cut off	1 point	2 points	4 points	6 points
Alcohol (ethanol) ^a^	0.01	0.05–0.10	0.11–0.15	0.16–0.25	≥0.26
Illicit drugs					
Amphetamines^b^	4/4.5	70–270	271–554	555–1052	≥1053
Cannabis (tetrahydrocannabinol)	0.15	0.6–2.8	2.9–4.7	4.8–9.7	≥ 9.8
Cocaine	6	60–243	244–515	516–789	≥ 790
Medicinal drugs					
Alprazolam	5	15–46	47–97	98–147	≥ 148
Clonazepam	5	9–47	48–98	99–148	≥ 149
Codeine	3	90–286	287–603	604–920	≥ 921
Diazepam	29	85–313	314–654	655–996	≥ 997
Flunitrazepam	0.8	1.6–6	7–12	13–18	≥ 19
Methadone	30	123–433	434–896	897–1360	≥ 1361
Morphine	3	15–54	55–140	141–284	≥ 285
Nitrazepam	7	14–53	54–110	111–166	≥ 167
Oxazepam	143	583–1118	1119–2265	2266–3411	≥ 3412
Zolpidem	8	46–104	105–212	213–430	≥ 431
Zopiclone	10	35–66	67–136	137–206	≥ 207

^a^ Values given in %. All other values are calculated and rounded off from original micromolar values, and given in ng/ml

^b^ Sum of amphetamine and/or methamphetamine

Time and place of injury, gender and age were taken from the questionnaire filled in at time of admittance to the hospital, whereas type of injury was found in the medical records from the hospital.

### Estimation of blood concentrations at time of injury

**Fig. 1:**
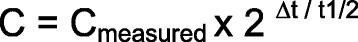
Back-calculation of drugs according to first-order kinetics, where C is the back-calculated concentration, C_measured_ is the measured concentration, ∆t the time span (in this case between injury and blood sampling), and t ½ the mean terminal half-life

To be able to assess the degree of influence of alcohol and impairing substances at the time of injury, the concentrations of alcohol and the different substances in blood at the time of injury were calculated. For alcohol, the concentration at the time of injury was estimated using a mean elimination rate of 0.015 % per hour multiplied by time passed from injury to blood sampling. For the other substances, a back-calculation was done using the measured concentration (C_measured_) multiplied by 2 elevated in the time span (∆_t_) divided by the half-life (t_1/2_) of the substance (Fig. 1). Half-lives of the different substances were taken from the mean value of the half-lives given in Baselt [13]. For substances having a mean half-life of 24 h or more (diazepam, methadone, nitrazepam and tetrahydrocannabinol), the measured concentrations were considered to reflect the concentrations at time of injury. Therefore a back-calculation of these concentrations to the time of injury was not done.

### Assessment of influence of alcohol and other impairing substances

The degree of influence of alcohol and impairing substances at the time of injury was assessed on the basis of the blood concentrations of substances and alcohol calcutated as described above. The degree to which different substances will influence a person’s behaviour will vary between individuals, but for several substances a positive relationship between blood concentration and drug influence has been found. These include benzodiazepines [8], zopiclone and zolpidem [14], amphetamines [15], tetrahydrocannabinol (THC) [16] and codeine [17]. Alcohol, as well as benzodiazepines and cannabis, is well studied concerning cognitive and psychomotor impairment and increased accident risk [18–20].

The impairing substances found in the present study were both medicinal substances which may be prescribed or used illegally, and illicit street drugs, as well as alcohol. A medicinal substance which is found in high non-therapeutic concentrations may indicate illicit use or drug abuse. It is important to differentiate between therapeutic use and non-therapeutic use of larger doses leading to influence when assessing the association between substance use and injuries. We gave the concentrations of alcohol and other impairing substances one to six points according to four concentration intervals for each substance (Table 1). Legal substances were scored so that one point corresponded to the average peak blood concentrations after ingestion of the maximum recommended therapeutic dose, for example 5–10 mg of diazepam or zopiclone, while increasing supratherapeutic concentrations corresponded to two, four or six points. Ethanol and illegal substances were scored in the same way, with intervals giving a degree of impairment comparable to the aforementioned drugs. In patients where more than one substance scored one point or more, the scores of the different substances were summed to give a total score, and a corresponding degree of influence. Patients who scored only one point were considered likely to be influenced by alcohol or substances, a total score of two to four points was taken to imply moderate influence, whereas a total score of five points or more was taken to imply marked influence. Comparing the different degrees of influence to BAC, 1 point or likely influenced will reflect a BAC of approximately 0.05–0.1 %, 2–4 points or moderate influence will reflect a BAC of approximately 0.1–0.2 %, and five or more points or marked influence will reflect a BAC around 0.2 % or even higher.

In Norway, impairment-based legislative limits are established for most of the substances included in the present study. The legislative limits, representing substance concentrations in blood likely to be accompanied by an impairment of driving skills comparable to blood alcohol concentrations of 0.02, 0.05 and 0.12 % [21], are low compared to the concentration intervals applied in this study. These differences are to a large extent due to the fact that the concentration intervals in the present study should apply to individuals who regularly use the different substances, and who might have developed tolerance to many of the substance effects, whereas the legislative limits are based on acute intake of substances in naïve individuals.

### Statistical analyses

Associations between degree of influence and patient and injury characteristics were analyzed in bivariate cross-tables. Pearson’s chi square test was used to assess categorical data. The dataset was checked for normality, and the independent sample *t*-test was used for comparison of means. Statistical analyses were performed using SPSS software (SPSS version 20). Level of significance was set at *p* < 0.05.

### Ethics

All patients who were invited to participate were informed about the project verbally as well as in writing and were asked to give a written informed consent. Patients were also informed that they could withdraw from the project at any time during data collection. The Data Inspectorate and the Regional Ethics Committee in Norway approved the study.

## Results

Two thousand seven hundred seventy-nine injured patients were admitted to Oslo university hospital over a 1 year period, of whom 2118 patients were asked to participate in the study. The 661 patients not asked to participate left the hospital before they were approached, were excluded due to language problems or mental status, or died. Apart from 158 patients who refused to participate, and 78 patients for whom there was no successful blood sampling, 1074 patients with a complete dataset arrived to the hospital within 6 h of injury. Of these 1074 patients, we excluded 78 patients who were admitted to the hospital due to acute poisoning, leaving us with a total of 996 patients.

Of the 996 patients included in the study, 36.9 %, or 394 of the patients, had one or more substances detected in blood at the time of admittance to the hospital. Of these, 324 (32.5 % of the total) were determined to be under the influence of one or more substances at the time of injury, that is, scoring at least 1 point using the criteria given in Table 1. Thus, approximately 82 % of the patients with one or more substances detected in blood at the time of admittance to the hospital were determined to be under the influence of one or more substances at the time of injury.

**Fig. 2:**
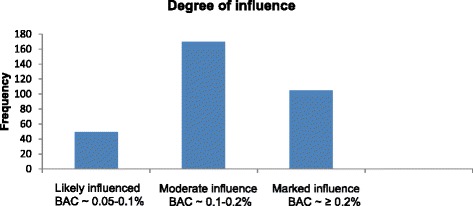
Distribution of patients in the different degrees of influence

The distribution of patients in the different categories of influence is given in Fig. 2. A total of 49 patients (15.1 %) were likely influenced, whereas the number of patients in the categories of moderate and marked influence were 170 (52.5 %) and 105 (32.4 %), respectively. The mean score of points in patients who were in the moderate influence category was 3.4 (95 % CI: 3.3–3.5), whereas the mean score of points in the marked influence group was 6.3 (95 % CI: 6.1–6.6). In the likely influenced category, the mean score of points was of course 1. The number of substances found in blood in patients who were not influenced ranged from 0 to 3, as compared to 1 to 8 substances in patients who were determined to be under the influence. In patients who were not influenced, the median of the number of substances, and interquartile range, was 0, whereas in influenced patients, the median of the number of substances, and interquartile range, was 1. There was an increasing number of substances with increasing degree of influence; 1–3 different substances in the likely influenced group, 1–6 different substances in the moderate influence group, and 1–8 different substances in the marked influence group.

Alcohol was the most prevalent substance, found over the cut-off limit of 0.01 % in 26.4 % of the patients at time of admittance to the hospital, and the substance most often causing influence, being found in a concentration of 0.05 % or higher in 25.9 % of the patients at the time of injury. Benzodiazepines were the second most prevalent substance group at 9.7 %, with 6.8 % in the influence range, whereas tetrahydrocannabinol was the third most prevalent substance at 5.8 % (4.7 % in the influence range). Approximately 98 % of the patients with alcohol detected over cut-off limit in blood at the time of admittance to the hospital were determined to be under the influence of alcohol at the time of injury, having a BAC of 0.05 or higher. Among patients with illicit drugs detected in blood at the time of admittance to the hospital, the proportion determined to be under the influence at the time of injury was about 79 %, whereas in patients with medicinal drugs detected in blood at the time of admittance to the hospital the proportion determined to be under the influence at the time of injury was 47 % (Table 2).

**Table 2:** Prevalence of the different substances above the cut-off limit at the time of admittance to the hospital, and percentage under the influence (comparable to a BAC of 0.05 % or higher) at the time of injury among patients who have the different substances in blood above the cut-off limit, and mean score of points at the time of injury

Substance	Prevalence n (%)	Influenced %	Mean score of points (95 % CI)
Alcohol	263 (26.4)	98.1	4.3 (4.1–4.5)
Medicinal drugs	163 (16.4)	47.2	4.3 (3.6–4.9)
Benzodiazepines	97 (9.7)	70.1	4.6 (3.9–5.4)
Diazepam	34 (3.4)	85.3	5.3 (4.2–6.3)
Clonazepam	17 (1.7)	88.2	5.4 (4.5–6.5)
Flunitrazepam	16 (1.6)	87.5	6.3 (4.6–8.0)
Oxazepam	16 (1.6)	75.0	5.4 (3.6–7.3)
Nitrazepam	12 (1.2)	83.3	3.5 (1.9–5.1)
Alprazolam	7 (0.7)	100.0	6.4 (3.9–9.0)
Zopiclone	35 (3.5)	40.0	2.4 (1.1–3.7)
Zolpidem	6 (0.6)	100.0	4.0 (1.5–6.5)
Opioids	44 (4.4)	40.9	4.3 (2.6–6.0)
Codeine	34 (3.4)	23.5	1.4 (0.6–2.1)
Morphine	11 (1.1)	81.8	6.6 (4.4–8.8)
Methadone	6 (0.6)	40.9	8.3 (5.3-11-2)
Illicit drugs	82 (8.2)	79.3	4.75 (4.1–5.4)
Tetrahydrocannabinol	58 (5.8)	81.0	4.3 (3.6–4.9)
^a^Amphetamines	18 (1.8)	88.9	5.6 (4.0–7.3)
Cocaine	11 (1.1)	63.6	6.0 (4.5–7.5)

^a^ Amphetamine and/or methamphetamine

Among the 324 patients who were determined to be under the influence of one or more substances, the total score of points ranged from one to twelve, with a mean (and median) score of four points. The only groups of patients who had a mean score of points below three were the oldest patients (above 65 years of age) and patients injured at place of work (Table 3).

**Table 3:** Prevalence of any substance at time of admittance to the hospital, and substance influence as well as mean score of points by gender, age, place of injury, type of injury, day of week and time of day at time of injury

	n (%)	Prevalence %	Influence %	Mean score of points in influenced patients (95 % CI)
Gender				
Female	378 (38)	34.9	22.5	3.5 (3.0–4.0)
Male	618 (62)	42.4	38.7	4.2 (3.9–4.4)
Age				
18–35	349 (35)	48.1	47	3.8 (3.5–4.1)
36–64	388 (39)	34.0	29.4	4.8 (4.4–5.2)
≥65	259 (26)	36.3	17.8	2.9 (2.2–3.5)
Place of injury				
Home	188 (18.9)	46.3	31.4	3.3 (2.7–3.9)
Bar/café/restaurant	43 (4.3)	88.4	88.4	4.2 (3.6–4.8)
Place of work	84 (8.4)	8.3	4.8	2.3 (0.4–4.1)
Other place	205 (20.6)	37.6	31.7	4.1 (3.5–4.7)
Street	460 (46.2)	38.3	32.6	4.2 (3.9–4.5)
Type of injury				
Violence	125 (12.6)	76.0	74.4	3.9 (3.5–4.3)
Fall	487 (48.9)	40.7	30.8	4.0 (3.7–4.4)
Other	384 (38.6)	26.3	21.1	4.1 (3.6–4.6)
Day of week				
Weekday	528 (53)	29.5	21.6	4.0 (3.5–4.4)
Weekend	468 (47)	50.9	44.9	4.0 (3.8–4.3)
Time of day				
Daytime (08–20)	594 (59.6)	23.4	14.0	3.8 (3.2–4.4)
Nighttime (20–08)	402 (40.4)	63.4	60.0	4.1 (3.8–4.3)

### Demographics

62 % of the patients included in the study were men, 38 % women. Their ages ranged from 18 to 99 years, with 35 % between 18 and 35 years, 39 % between 36 and 64 years, and 26 % above 65 years. The distribution of the different categories of influence differed statistically significantly by age and with gender. Men were statistically significantly (*p* < 0.001) more often under the influence (any category) than women, with almost 40 % determined to be under the influence compared to a little over 20 % of the women (Table 3). Considering the total score of points in patients who were under the influence, the difference between men and women was statistically significant. The mean score in men under the influence was 4.2 (95 % CI: 3.9–4.4) points, while the mean score in women under the influence was 3.5 (95 % CI: 3.0–4.0, *p* < 0.05). Patients who were under the influence were younger, with a mean age of 40.8 (95 % CI: 38.6–43) versus 52.9 (95 % CI: 51.2–54.6, *p* < 0.001) for patients who were not influenced.

### Type and place of injury

In patients with violence-related injuries, almost 75 % were determined to be under the influence of one or more substances, compared to injuries due to falls or other causes, where the proportion of patients who were determined to be under the influence was approximately 30 and 20 %, respectively (Table 3). None of the patients over 65 years of age with violence-related injuries were under the influence. Patients admitted with violence-related injuries were younger (*p* < 0.001), as concerns both those who were under the influence, with a mean age of 28.6 (95 % CI: 27–30.2), and those who were not influenced, with a mean age of 37.6 (95 % CI: 32.6–342.6), compared with patients suffering non-violent injuries, who had a mean age of 45.7 (95 % CI: 43.1–48.3) for those who were under the influence and 53.6 (95 % CI: 51.8–55.5) for those who were not influenced. In road traffic accidents, 20 % of the drivers were under the influence, with a mean score of 4.4 (95 % CI: 3.2–5.6) points. Looking at place of injury, nearly 90 % of the patients injured in bars, cafés or restaurants were under the influence. The mean score among these patients was 4.2 (95 % CI: 3.6–4.8) points. As expected, only a few patients, less than 5 %, were under the influence when injured at a place of work.

### Time of day and day of week

More patients were under the influence at nighttime, and at the weekend (Table 3). 75 % of the patients admitted at nighttime at the weekend were determined to be under the influence, as compared to only about 12 % in daytime on weekdays. Patients admitted at nighttime who were under the influence were younger, with a mean age of 37.6 (95 % CI: 35.4–39.8) versus 47.5 (95 % CI: 43.9–51.1, *p* < 0.001) for patients admitted at nighttime considered not to be influenced.

### Multivariable analysis

A multivariable analysis was conducted to assess which patient characteristics were associated with being under the influence of any psychoactive substance (Table 4). The analysis showed that male gender was significantly associated with being under the influence (OR 2.2, 95 % CI: 1.6–2.9), whereas increasing age was associated with less influence. Violence-related injuries, and injuries occurring at weekends and at nighttime, yielded the highest odd ratios for being under the influence. All of the above-mentioned characteristics were statistically significant in both univariate and multivariable analysis.

**Table 4:** Odds ratios from univariate and multivariable analysis examining the relationship between injury during substance influence and gender, age, type of injury, day of week and time of day

	Univariate analysis		Multivariable analysis	
Factors	OR	95 % CI	OR	95 % CI
Gender				
Female	1		1	
Male gender	2.2	1.6–2.9	1.6	1.1–2.3
Age				
Age 18–35	1		1	
Age 36–64	0.5	0.4–0.6	0.7	0.5–1.0
≥ 65	0.2	0.2–0.4	0.4	0.3–0.7
Type of injury				
Other	1		1	
Fall	1.7	1.2–2.3	2.6	1.7–3.7
Violence	10.9	6.8–17.4	5.3	3.1–9.0
Day of week				
Weekday	1		1	
Weekend	3.0	2.2–3.9	2.7	2.0–3.8
Time of day				
Daytime (08–20)	1		1	
Nighttime (20–08)	9.2	6.8–12.5	6.4	4.6–9.0

## Discussion

To our knowledge, this study is the first where blood concentrations of alcohol and other impairing substances at the time of injury are calculated, and also the first where an assessment of the degree of substance influence at the time of injury has been performed. The results showed that about one third of the patients included, numbering 324 or 32.5 %, were under the influence of one or more substances at the time of injury, with alcohol being the most prevalent substance causing influence. Approximately 85 % of the patients who were determined to be under the influence were in the categories of moderate or marked influence, having a BAC, or substance influence similar to a BAC, higher than 0.1 %.

Among patients with alcohol above the cut-off limit in blood at time of admittance to the hospital, 98.1 % were determined to be under the influence of alcohol at the time of injury, whereas approximately 80 % of patients with illicit drugs in blood at the time of admittance to the hospital were under the influence of illicit drugs at the time of injury. Patients with medicinal substances in blood had the lowest proportion determined to be under the influence of these substances at the time of injury at about 50 %. Still, the fact that almost half the patients with medicinal substances in blood were under the influence constitutes a fairly high share, indicating that abuse of medicinal substances is widespread.

For medicinal substances, there was a large gap between the lowest and highest percentage of patients determined to be under the influence of the different substances at the time of injury compared to prevalence at the time of admittance to the hospital. It ranged from approximately 23 % for codeine, to 100 % for alprazolam and zolpidem, indicating that codeine is used to a large extent in a therapeutic manner, compared to, for instance, alprazolam, which is a widely abused substance in Norway. This is also reflected in the mean score of points and mean number of substances in patients influenced by the different substances, which for codeine was 1.4 points and 1.8 substances, versus 6.4 points and 4.7 substances for alprazolam.

A strength of our study is the fact that we calculated the concentrations of the different substances in blood at the time of injury. This is especially important for substances with a short half-life, like zopiclone, zolpidem and morphine. However, we did not make an estimation of tetrahydrocannabinol concentrations at the time of injury, since its half-life largely depends on time elapsed from intake [22, 23]. This may be a limitation, since tetrahydrocannabinol generally has a short half-life, and it also was the third most prevalent substance causing influence. Also, substances with short half-lives may have been present in blood in considerable concentrations at the time of injury, and still come under cut-off limits at the time of admittance to the hospital up to 6 h later.

Concerning the assessment of degree of influence, this was made solely on the concentrations of the different substances in blood. This is a limitation, since the assessment preferably should be supported by a clinical evaluation of signs and symptoms of influence, as is generally done in forensic toxicology. However, a positive relationship between blood concentrations and influence in apprehended drivers has been established for many of the substances included in this study.

In our study, patients who were under the influence had a larger number of substances in blood, ranging from 1 to 8 substances, compared with patients who were not influenced, who only had 0 to 3 substances in blood. The number of substances in patients under the influence also increased with increasing degree of influence. When looking at traffic accident risk, several studies have found that the combination of several substances dramatically increases accident risk [24, 25]. This increased risk will probably also apply to injuries in general, and may additionally have contributed to the risk of injury in our group of patients under the influence, who generally had several different substances in blood.

Several previous studies have found a significant relationship between alcohol and drug use and violence-related injuries [2, 26]. These types of injuries stand out in our material as well, with almost 75 % being under the influence at time of injury. The highest rates of influence related to injury were not surprisingly also found for violence-related injuries.

## Conclusions

In conclusion, about one third of the patients admitted with injuries were determined to be under the influence at the time of injury, with alcohol being the most prevalent substance causing influence. Male gender was significantly associated with being under the influence at the time of injury, whereas increasing age was associated with less influence. Violence-related injuries, and injuries occurring at nighttime and during weekends appeared, in particular, to be related to substance influence. Approximately 98 % of the patients with alcohol detected above the cut-off limit in blood at the time of admittance to the hospital were under the influence of alcohol at the time of injury, having a BAC of 0.05 % or higher. Among patients with illicit and medicinal substances detected in blood at the time of admittance to the hospital, the proportion determined to be under the influence of these substances at the time of injury were 79 and 47 %, respectively. The results from our study suggest that detection of alcohol in blood at the time of admittance to the hospital indicates alcohol influence at the time of injury. For medicinal substances, and to some degree for illicit drugs, the association between detection in blood at the time of admission to the hospital and substance influence at the time of injury is less clear.

## Abbreviations

∆_t_, time span; BAC, blood alcohol concentration; CI, confidence interval; C_measured_, measured concentration; OR, odds ratio; t_1/2_, half- life; THC, tetrahydrocannabinol

## References

[1] Perez K, Santamarina-Rubio E, Rodriguez-Martos A, Brugal MT, Ricart I, Suelves JM (2009). Substance use among non-fatally injured patients attended at emergency departments in Spain. Drug Alcohol Depend.

[2] Vitale S, van de Mheen D (2006). Illicit drug use and injuries: A review of emergency room studies. Drug Alcohol Depend.

[3] Cherpitel CJ (2007). Alcohol and injuries: a review of international emergency room studies since 1995. Drug Alcohol Rev.

[4] Bogstrand ST, Normann PT, Rossow I, Larsen M, Morland J, Ekeberg O (2011). Prevalence of alcohol and other substances of abuse among injured patients in a Norwegian emergency department. Drug Alcohol Depend.

[5] Verstraete AG (2004). Detection times of drugs of abuse in blood, urine, and oral fluid. Ther Drug Monit.

[6] Wolff K, Farrell M, Marsden J, Monteiro MG, Ali R, Welch S (1999). A review of biological indicators of illicit drug use, practical considerations and clinical usefulness. Addiction.

[7] Dubowski KM (1985). Absorption, distribution and elimination of alcohol: highway safety aspects. J StudAlcohol Suppl.

[8] Bramness JG, Skurtveit S, Morland J (2002). Clinical impairment of benzodiazepines--relation between benzodiazepine concentrations and impairment in apprehended drivers. Drug Alcohol Depend.

[9] Mordal J, Bramness JG, Holm B, Morland J (2008). Drugs of abuse among acute psychiatric and medical admissions: laboratory based identification of prevalence and drug influence. Gen Hosp Psychiat.

[10] Jones AW (2010). Evidence-based survey of the elimination rates of ethanol from blood with applications in forensic casework. Forensic SciInt.

[11] Verstrate AKA, Jantos R, Skopp G, Gjerde H, Vindenes V, Morland J, Langel K, Lillsunde P (2011). Per se limits - Methods of defining cut-off values for zero tolerance.

[12] Morland J (2000). Driving under the influence of non-alcohol drugs. Forensic Sci Rev.

[13] Baselt RC. Disposition of toxic drugs and chemicals in man. 9 ed. Seal Beach, California: Biomedical publications; 2011.

[14] Gustavsen I, Al-Sammurraie M, Morland J, Bramness JG (2009). Impairment related to blood drug concentrations of zopiclone and zolpidem compared to alcohol in apprehended drivers. AccidAnal Prev.

[15] Gustavsen I, Morland J, Bramness JG (2006). Impairment related to blood amphetamine and/or methamphetamine concentrations in suspected drugged drivers. AccidAnal Prev.

[16] Khiabani HZ, Bramness JG, Bjorneboe A, Morland J (2006). Relationship between THC concentration in blood and impairment in apprehended drivers. Traffic InjPrev.

[17] Bachs L, Skurtveit S, Morland J (2003). Codeine and clinical impairment in samples in which morphine is not detected. Eur J Clin Pharmacol.

[18] Blomberg RD, Peck RC, Moskowitz H, Burns M, Fiorentino D (2009). The Long Beach/Fort Lauderdale relative risk study. JSafety Res.

[19] Taylor B, Irving HM, Kanteres F, Room R, Borges G, Cherpitel C (2010). The more you drink, the harder you fall: a systematic review and meta-analysis of how acute alcohol consumption and injury or collision risk increase together. Drug Alcohol Depend.

[20] Drummer OH, Gerostamoulos J, Batziris H, Chu M, Caplehorn J, Robertson MD (2004). The involvement of drugs in drivers of motor vehicles killed in Australian road traffic crashes. AccidAnalPrev.

[21] Vindenes V, Jordbru D, Knapskog AB, Kvan E, Mathisrud G, Slordal L (2012). Impairment based legislative limits for driving under the influence of non-alcohol drugs in Norway. Forensic SciInt.

[22] Huestis MA (2007). Human cannabinoid pharmacokinetics. ChemBiodivers.

[23] Toennes SW, Ramaekers JG, Theunissen EL, Moeller MR, Kauert GF (2008). Comparison of cannabinoid pharmacokinetic properties in occasional and heavy users smoking a marijuana or placebo joint. JAnalToxicol.

[24] Gjerde H, Normann PT, Christophersen AS, Samuelsen SO, Morland J (2011). Alcohol, psychoactive drugs and fatal road traffic accidents in Norway: a case-control study. AccidAnalPrev.

[25] Kuypers KP, Legrand SA, Ramaekers JG, Verstraete AG (2012). A case-control study estimating accident risk for alcohol, medicines and illegal drugs. PLoSOne.

[26] Cunningham R, Walton MA, Maio RF, Blow FC, Weber JE, Mirel L (2003). Violence and substance use among an injured emergency department population. AcadEmergMed.

